# Electrical control of quantum emitters in a Van der Waals heterostructure

**DOI:** 10.1038/s41377-022-00877-7

**Published:** 2022-06-20

**Authors:** Simon J. U. White, Tieshan Yang, Nikolai Dontschuk, Chi Li, Zai-Quan Xu, Mehran Kianinia, Alastair Stacey, Milos Toth, Igor Aharonovich

**Affiliations:** 1 School of Mathematical and Physical Sciences, University of Technology Sydney, Ultimo, NSW 2007 Australia; 2 ARC Centre of Excellence for Transformative Meta-Optical Systems, University of Technology Sydney, Ultimo, NSW 2007 Australia; 3 School of Physics, University of Melbourne, Parkville, VIC 3010 Australia; 4 School of Science, RMIT University, Melbourne, VIC 3001 Australia

**Keywords:** Optics and photonics, Optical materials and structures

## Abstract

Controlling and manipulating individual quantum systems in solids underpins the growing interest in the development of scalable quantum technologies. Recently, hexagonal boron nitride (hBN) has garnered significant attention in quantum photonic applications due to its ability to host optically stable quantum emitters. However, the large bandgap of hBN and the lack of efficient doping inhibits electrical triggering and limits opportunities to study the electrical control of emitters. Here, we show an approach to electrically modulate quantum emitters in an hBN-graphene van der Waals heterostructure. We show that quantum emitters in hBN can be reversibly activated and modulated by applying a bias across the device. Notably, a significant number of quantum emitters are intrinsically dark and become optically active at non-zero voltages. To explain the results, we provide a heuristic electrostatic model of this unique behavior. Finally, employing these devices we demonstrate a nearly-coherent source with linewidths of ~160 MHz. Our results enhance the potential of hBN for tunable solid-state quantum emitters for the growing field of quantum information science.

## Abstract (web summary)

The image depicts a schematic illustration of a van der Waals heterostructure used to electrically activate quantum emitters in hexagonal boron nitride.

## Graphical abstract

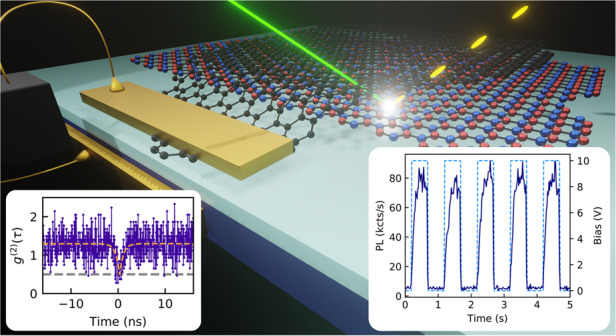

## Introduction

Van der Waals (vdW) heterostructures have emerged as a fascinating platform to study light-matter interaction at the nanoscale^1–4^. Assembling various atomically thin crystals has enabled the observation of new physical phenomena in these unconventional materials, including superconductivity^5^, interlayer excitons^6^, moire lattices^1,7^, and correlated electronic systems^8^. Furthermore, advanced practical devices such as broadband photodetectors, efficient light-emitting diodes, and nanoscale lasers have also been realized from a variety of vdW crystals^9^. Indeed, control over light emission from a selected family of transition metal di-chalcogenides enabled optical detection of valley states, and observation of exciton-polariton condensates even at room temperature^10–13^

Of particular interest is the ability to manipulate light emission from single-point defects, commonly referred to as single-photon emitters (SPEs), as they are critical building blocks for quantum technologies^14,15^. Hexagonal boron nitride (hBN), a wide bandgap vdW crystal, has been extensively studied in recent years as a vdW host of SPEs that are ultra-bright and optically stable^16–20^. In addition, hBN SPEs exhibit spin–photon interface and can be engineered on demand in an atomically thin crystal^21,22^. This combination of photophysical properties foreshadows ample opportunities for their utilization as quantum sources and quantum repeaters in scalable quantum photonic devices. An outstanding challenge for solid-state SPEs is to realize electrical control of the optical emission. This challenge stems from the fact that most hosts of defect-based SPEs are wide bandgap materials in which p-type or n-type doping is limited^23,24^. Indeed, even for well-studied materials such as diamond or silicon carbide, electrical modulation of quantum emitters is limited to specific defects and often requires cumbersome device engineering^25–28^.

## Results

Here we demonstrate a facile and scalable approach to electrically modulate quantum emitters in hBN-graphene heterostructures. Our experiments show that SPEs in hBN can be controllably activated and modulated by applying a voltage across the devices. Intriguingly, we show that most of the quantum emitters become optically active at non-zero voltages, in contrast to what has been observed in the case of defects in 3D crystals. We interpret our results in the context of electrically-induced changes in the charge states of the hBN defects and provide electrostatic models to support the experimental findings.

**Fig. 1:**
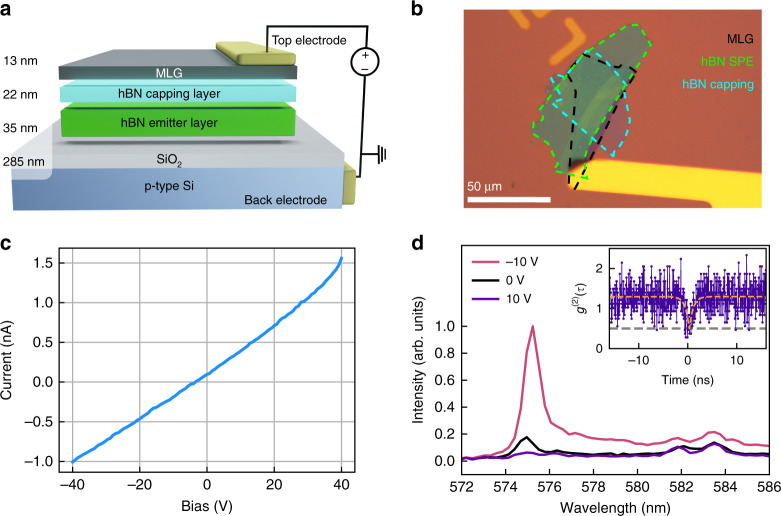
The hBN/MLG heterostructure device. **a** Schematic of the device and its operating principle. The device is biased using gold electrodes in contact with MLG and p-type silicon. Layer thicknesses are indicated on the left. **b** Optical image of a device consisting of MLG, an hBN capping layer, and an hBN layer that contains SPEs. The substrate is bulk p-type silicon with a 285 nm thermal oxide layer. Each layer is outlined by dashed lines. **c** *I*–*V* curve from the device shows a negligible leakage current. **d** Normalized PL spectra of a fluorescence peak at 575 nm from the heterostructure under a bias of −10 V (red), 0 V (black), and 10 V (violet), at 4 K. Inset: Second-order correlation data measured from the same emitter under −10 V bias, at 4 K. The PL was filtered using a tunable bandpass filter centered at 575 ± 2 nm and the fit (dashed yellow) reveals *g*^(2)^(0) = 0.48. The *g*^(2)^(t) model and analysis are developed further in the supplementary material

Figure 1a is a schematic illustration of the heterostructure devices used in this study. The device structure consists of multilayer graphene (MLG), an hBN capping layer, and an hBN emitter layer stacked vertically on p-type silicon with a 285 nm thermal oxide. Bias is applied between the bottom p-type silicon and MLG. An optical image of the device is shown in Fig. 1b. The black, light blue, and green dashed lines indicate the boundaries of the MLG, the hBN capping layer, and the hBN layer that hosts the quantum emitters, respectively. The capping layer (~20 nm) is used to prevent the quenching of emitters in the active hBN layer by MLG^29^. Details of the fabrication process can be found in the methods section.

To characterize the device, we first measured a current–voltage (*I–V*) curve by sweeping the bias from −40 V to 40 V. The current scales linearly with voltage, as shown in Fig. 3c, and the maximum measured current is less than 1.5 nA. This is an upper bound on the current through the hBN layers since the top electrode is in contact with both the MLG and the oxide layer (see Fig. 1b). The *I–V* curve shows that the device behaves as a capacitor that generates an electric field within the hBN layers. Additional electrical measurements from the device are included in the supporting information.

Next, we study the optical properties of quantum emitters embedded in the heterostructure. Almost all optical measurements were performed using a 532 nm continuous-wave excitation laser, and a custom-built confocal microscope (see methods for details); some exceptions are noted within the text. To elucidate this peculiar behavior, the hBN/MLG heterostructure device was loaded into a closed cycle He cryostat operating at 4 K. Figure 1d shows photoluminescence (PL) spectra from one emitter at 4 K, using a bias of −10 V (red curve), 0 V (black curve) and 10 V (violet curve). Remarkably, a clear peak at ~575 nm arises when the voltage is switched from 10 V to 0 V and increased further at −10 V as shown in Fig. 1d, indicating activation of the emitter by the applied bias. Additional spectra are presented in the SI. The switching behavior also persisted at room temperature (Fig. S5). After filtering the emission peak at 575 nm using a 575 ± 2 nm bandpass filter, second-order correlation measurements were performed. A dip at zero delay, *g*^(2)^(0) = 0.48, indicates the presence of nonclassical emission which we attribute to a single quantum emitter with some background PL. Bunching is observed above a few nanoseconds as the *g*^(2)^ data plateaus to a value of 1.3, which indicates the presence of an additional metastable state. Further second-order correlation analysis is available in the supplementary material. Here we note that as the background PL also varies with the applied bias, no background correction was used for *g*^(2)^(0) measurements, thus the *g*^(2)^(0) value represents an upper bound for these emitters (detailed in the Fig. S11).

**Fig. 2:**
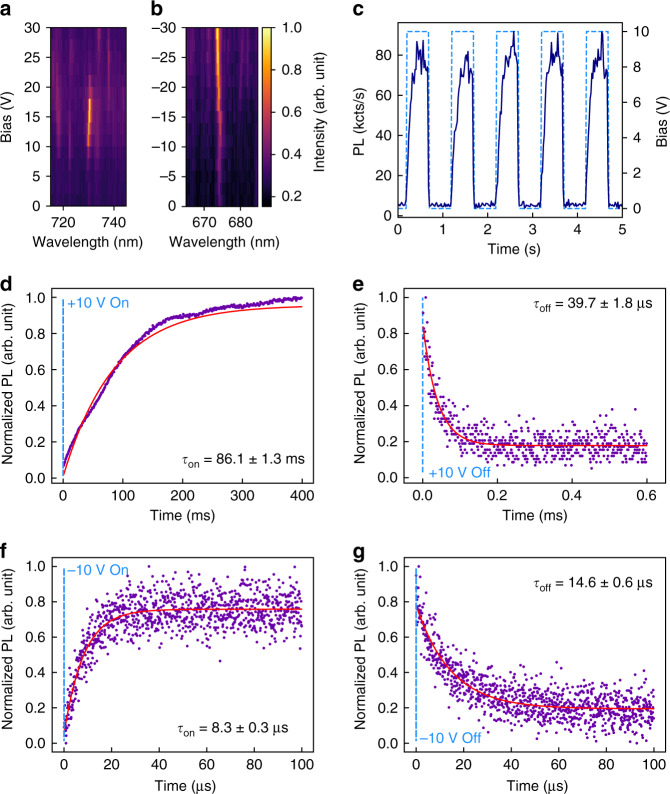
Electrical control of hBN emitters in the heterostructure device. **a**, **b** Normalized PL spectra recorded from two different emitters as the bias applied to MLG varied from 0 V to + 30 V (**a**), and −30 V (**b**). **c** Dynamic modulation of the emission intensity of an emitter by a square wave bias function. The bias is switched periodically between 0 V and + 10 V, as shown by the light blue trace. The filtered PL signal intensity detected by an avalanche photodiode (APD) is plotted in dark blue. **d**, **e** Normalized PL intensity versus time, showing the emission dynamics when the emitter is turned on (**d**) and off (**e**) by a + 10 V step function applied to the MLG electrode. The measured data are fitted with single exponential functions, and the time constants, τ_on,_ and τ_off_ are 86 ms and 40 μs, respectively. **f**, **g** Corresponding dynamics from an emitter that becomes active under negative bias, measured by applying a −10 V step function to the MLG electrode. Under negative bias, τ_on_ and τ_off_ are 8 μs and 15 μs, respectively

The electrical control of the hBN emitters is shown in Fig. 2. The bias dependence of PL spectra from two emitters is plotted in Fig. 2a, b. The spectra, normalized for clarity, illustrate two distinct behaviors observed predominantly under positive (Fig. 2a) and negative (Fig. 2b) bias applied to the MLG electrode. The emitter in Fig. 2a does not fluoresce at zero bias. However, as the bias is increased, the emitter becomes active at ~8 V, and increases in brightness up to ~15 V where it goes through a maximum and then decreases as the bias is increased further. It becomes inactive at ~22 V and is not returning to its optically active state as higher bias is applied (within our experimental limitations). On the other hand, the emitter in Fig. 2b shows completely different behavior. As the bias is reduced from 0 V to −30 V, the emission intensity increases gradually and remains optically active even under –30 V. This is unexpected, given that under positive biases, there was only a window of voltages under which the emission was persistent. This would be explained later in detail. Note, that in both cases, a minor shift of the emission was observed, as expected, due to the Stark shift^30,31^. The direction of the Stark shifts depends on the polarity of the applied bias and the dipole orientation of each emitter.

The emission intensity can be further tuned dynamically with the applied bias. This is shown in Fig. 2c, where an emitter is modulated using a square wave voltage function oscillating between 0 V and +10 V. The period of the intensity modulation resembles the square wave bias function, illustrating the repeatability of the activation process—the switching is reversible and repeatable. Similar behavior was also observed for emitters activated by a negative bias applied to the MLG (Fig. S14).

A detailed analysis of the switching rates is presented in Fig. 2d–g. The time-correlated intensity was recorded using a time tagger (Swabian instrument, jitter of <200 ps) whilst bias step functions were applied to the device. Figure 2d, e shows the PL rise and decay times when a bias of +10 V was turned on and off, respectively. The curves were fitted with single exponential functions and the rise (τ_on_) and fall times (τ_off_) are estimated to be ~86 ms and ~40 µs, respectively. The rise time is ~2000 times slower than the fall time, indicating significant differences between the charging and discharging dynamics ^32^.

The corresponding measurements obtained using a negative bias of −10 V are shown in Fig. 2f, g. Under negative bias, τ_on_ and τ_off_ are comparable, approximately 8 µs and 15 µs, respectively. Strikingly, the rise time under negative bias is over four orders of magnitude faster than under the positive bias, whilst the fall times are similar under both positive and negative bias. The dramatic difference between the rise times is indicative of distinct emitter activation mechanisms under positive and negative bias, as is discussed in detail below.

**Fig. 3:**
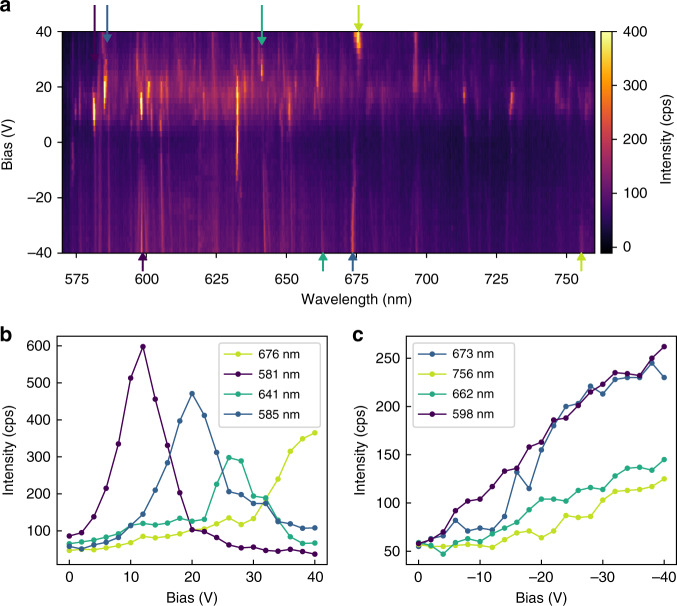
Activation of hBN emitters in the heterostructure device. **a** PL spectra recorded as a function of bias, over the range of −40 to + 40 V. The lines at 580 nm and 620 nm are the G band and 2D band of MLG. The remaining lines are emitters in hBN. The upper and lower arrows indicate the position of the PL lines from which peaks are emission maxima are extracted for (**b**) and (**c**) respectively. **b**, **c** Emission intensity versus bias for a number of emitters activated by a positive (**b**) and a negative (**c**) voltage applied to the MLG electrode

To provide a broad, statistically-representative overview of the behavior of emitters under applied bias, we recorded PL spectra from a large ensemble of emitters within the area of a single excitation laser spot. The spectra recorded as a function of bias over the range of −40 V to +40 V is shown in Fig. 3a, where each emission line corresponds to an emitter in hBN. The lines at 580 nm (620 nm) are the G (2D) bands of MLG and remain unchanged (at this particular spectrometer resolution using a 300 lines/mm grating)^33^.

A large number of emitters spanning a broad range of emission wavelengths are activated when a positive bias is applied to the MLG electrode, mostly above +10 V. Similarly, numerous emission lines appear when a negative bias is applied to the device, and become increasingly brighter as the bias decreases to −40 V. We note that no emission was observed from the device at any bias in the absence of the excitation laser—that is, all emissions discussed in this paper are field-activated PL rather than electroluminescence.

To investigate this effect further, we plot the intensity from a number of representative emitters as a function of applied bias in Fig. 3b, c. Figure 3b shows four emissions that are active within a positive bias range. The peak intensity at each chosen wavelength is extracted from the maximum intensity over 1.5 nm range. This window also helps account for spectral wandering and any stark shift. The PL intensity from each of these emitters is highly bias-dependent. For example, the intensity of the 581 nm line peaks at a bias of ~10 V, while the 641 nm line peaks at ~28 V. Interestingly, most of the emitters have a clear bias activation range—that is, they are optically active over this range and inactive at biases outside this range. Such behavior has never been observed for any other solid-state quantum emitters, and it is discussed in detail below.

The behavior is substantially different when a negative bias is applied to the MLG electrode. As is shown in Fig. 3a, as the bias is reduced from 0 V to −40 V, a number of emitters become optically active and none of them deactivate over the entire bias range. The intensity of a number of representative emissions from this group is plotted versus bias in Fig. 3c. The emitters are very dim at zero bias, and the emission intensities increase linearly as the bias is reduced from 0 V to −40 V under constant laser excitation power. We note that an increase in emitter intensity versus bias has been observed previously for neutrally charged NV centers in diamond^25,34^. However, more broadly, the observation of PL emissions that are inactive until a voltage is applied has not been reported for any solid-state quantum systems. Finally, based on the above results, most of the emitters appear to be trackable from the positive to the negative voltage range, indicating that they are the same emitters (i.e., each spectral line corresponds to the same emitter—or emitters belong to the same crystallographic origin).

## Discussion

**Fig. 4:**
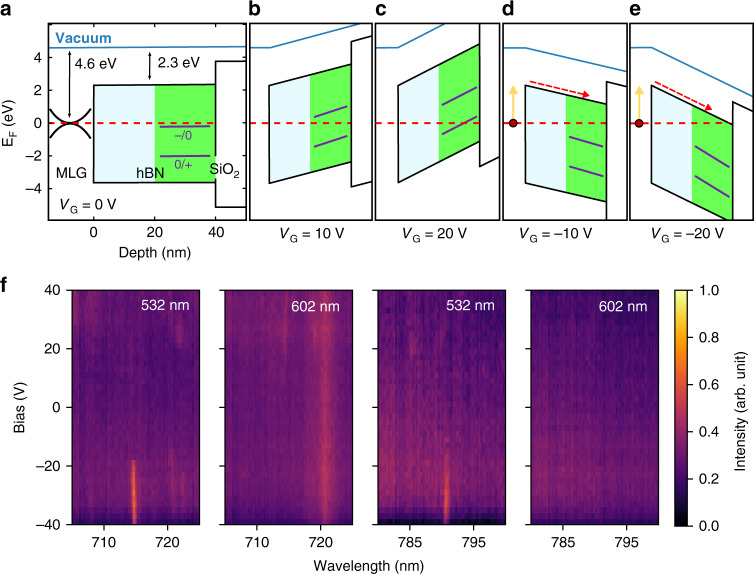
Band diagram of the heterostructure device under various bias configurations. **a** The device where both electrodes are grounded (*V*_G_ = 0 V). The hBN capping layer is shown in light blue and the hBN layer that contains quantum emitters is shown in green (the thickness of each hBN layer is assumed to be 20 nm). The MLG quasi-Fermi level (*E*_F_) extends into the hBN, indicating charge transfer between MLG and defect states in hBN (see text). The purple lines indicate two hypothetical charge transition levels of a single defect in hBN. **b**, **c** The device with a bias of +10 V (**b**) and +20 V (**c**) applied to the MLG electrode. **d**, **e** The device with a bias of −10 V (**d**) and −20 V (**e**) applied to the MLG electrode. The solid yellow arrows show photoexcitation of an electron in MLG, and broken red arrows indicate the drift of the electrons via the band gaps of the hBN layers. **f** Experimental verification of the model, whereby emitters (at ~715 nm and 790 nm) are only visible under green excitation (λ_exc_ = 532 nm) but not under lower energy red excitation (λ_exc_ = 602 nm), at the same confocal spot. The emission at 720 nm is graphene Raman^33^

We now turn to a discussion of the photophysics of these emitters under applied bias. We attribute the emitter activation and deactivation caused by a positive bias (seen in Fig. 3b) to changes in charge states of defects in hBN, and the activation of emitters under negative bias (seen in Fig. 3c) to the injection of hot electrons from MLG into hBN. These two processes are characterized by the slow and fast emitter activation dynamics, as is discussed below in the context of the electron energy level diagram shown in Fig. 4.

The device band diagram under zero bias is shown in Fig. 4a. The MLG quasi-Fermi level, *E*_F_, and the bottom of the hBN conduction band are located 4.6 eV and 2.3 eV below the vacuum level, respectively^35^. Also shown on the diagram are two hypothetical charge transition levels of a defect in hBN, adapted from reported density functional theory (DFT) calculations^29,36^. Fig. 4b, c shows the device at a bias of +10 V and +20 V, respectively, and illustrate how a positive bias sweep causes sloping of the energy bands, and an effective sweep of E_F_ within a subset of the bandgap of hBN. A defect with a charge transition level within this region of the bandgap will gain/lose an electron as E_F_ moves above/below the level (Fig. 4b). Similarly, a defect with two charge transition levels in this region of the bandgap will change charge state twice if *E*_F_ sweeps through both levels. Hence, the hBN defect in Fig. 4a will have lost two electrons upon the application of +20 V to the MLG (Fig. 4c). Each change in the charge state of an emitter will result in a corresponding change in the defect energy levels and hence the emission spectrum^25,34^. Importantly, a change in charge state often causes activation or deactivation of an emitter—either absolutely or effectively by causing the emission energy to shift outside the measured spectral range^25^. Hence, activation of an hBN quantum emitter upon the application of a positive bias to the MLG electrode of our heterostructure device can be caused by a change in the charge state of the emitter by +1 (Fig. 4b). Deactivation of the emitter at a greater positive bias can be caused by the second change in charge state, provided that E_F_ crosses a second charge transition level of the emitter (Fig. 4c). Note that the defects are located at various depths of the hBN layer. Hence, upon voltage application, the band bending would influence differently the different defects (and their corresponding charge transition levels) due to different distances from the graphene/p-doped silicon (discussed further below). A variation in the local environment of the defects can also account for the different voltages (and consequently the electric fields) required to control the emitters.

Based on the above, activation of an emitter upon application of a negative bias could be argued to be caused by a change in the charge state of the emitter by −1. However, an upward sweep of E_F_ within the bandgap of hBN will populate deep defect levels and we do not expect it to activate emitters. Moreover, we found that the activation rate measured by applying a step voltage function to the device is over three orders of magnitude slower for the case of positive bias than for the case of negative bias (Fig. 2d, f, respectively), indicating a fundamental difference in the charge transfer dynamics. To explain this difference, we consider energy band diagrams for the negatively charged device shown in Fig. 4d, e for the case of −10 V and −20 V, respectively. Application of a bias that is negative with respect to the MGL electrode inverts the gradient of the sloped bands and effectively raises *E*_F_ towards the hBN conduction band. In this configuration, electrons excited in the MLG by the laser (yellow arrows in Fig. 4) can tunnel across the barrier at the MLG–hBN interface and drift (red broken arrows) within hBN under the influence of the applied electric field. The resulting photocurrent provides a means to supply hot electrons to emitters *via* the hBN conduction band. This charge transfer mechanism is therefore expected to be fast relative to the case of a positive bias (Fig. 4b, c), where electron removal from the deep hBN charge transition levels likely occurs via a hopping mechanism and electrons flow to the MLG *via* trap states inside the hBN bandgap.

The above analysis illustrates two distinct charge transfer mechanisms between the MLG electrode and defects in hBN, which are slow/fast in the case of positive/negative bias applied to the MLG. The first can account for emitter activation and deactivation upon application of a positive voltage sweep to the device, and the second can account for emitter activation by a negative bias. We note that the almost universal deactivation of emitters at +40 V, seen in Fig. 3a, is likely a consequence of the fact that *E*_F_ lies very close to the hBN valence band and the ground states of most emitters are ionized at this voltage. We also note that, as is evident from Fig. 4, the voltage needed to activate/deactivate various emitters is a function of the emitter location within the hBN. This observation combined with the fact that a number of distinct defect species are responsible for the rich emission spectrum of hBN accounts for the variation in activation and deactivation voltages seen in Fig. 3a.

To provide further experimental support for our model, we increased the excitation laser wavelength from 532 nm (~2.3 eV) to 602 nm (~2 eV). The longer wavelength excitation should not be sufficient to overcome the energy barrier (see Fig. 4d, e) under negative bias, and hence no emitters should be activated. Indeed, this hypothesis is confirmed. Figure 4f shows PL spectra of emitters under positive and negative bias recorded at the same confocal spot using the two excitation wavelengths. It is clear that new emitters appear under negative bias when a 532 nm excitation laser is used, but no emission appears under the longer excitation wavelength of 602 nm.

**Fig. 5:**
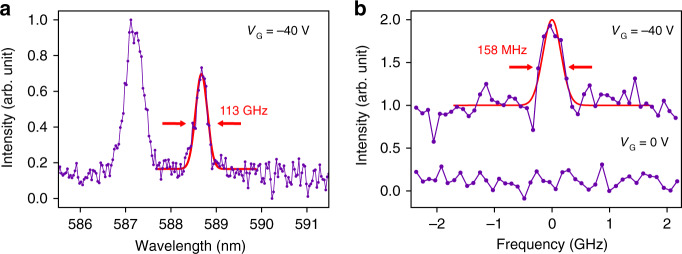
Coherent excitation of quantum emitters in hBN. **a** Emission spectrum of a single emitter with a ZPL at ~588.5 nm, recorded under non-resonant 532 nm excitation. **b** Resonant excitation of the same emitter, resulting in a nearly-coherent photon source with a linewidth of ~158 MHz. Both measurements were done using a bias voltage, *V*_G_ = –40 V

To illustrate the potential of our devices for practical and scalable quantum photonic applications, we demonstrate the resonant excitation of these quantum emitters under a negative bias. We expect that under these conditions, the charge transfer under bias governs the charge states of both emitters and surrounding charge traps and thus suppress charge fluctuations and spectral diffusion of quantum emitters under resonant excitation^27^. This was indeed observed, as is illustrated in Fig. 5. Figure 5a shows an emitter with a ZPL at ~588.5 nm, recorded from the device using an off-resonant 532 nm excitation laser. The off-resonant linewidth is phonon broadened as expected. Figure 5b shows a resonant excitation scan of the same emitter with a measured linewidth of ~158 ± 19 MHz. Both measurements were taken using an applied bias, *V*_G_ = −40 V, and importantly, no resonant emission was observed at zero bias. For quantum emitters in hBN with excited-state lifetimes on the order of ~3 ns, ~160 MHz certainly represents a nearly-coherent, Fourier Transform limited, linewidth, which is highly promising for future-generation indistinguishable photons.

To summarize, we demonstrate electrical modulation and control of a variety of quantum emitters in a vdW heterostructure. The quantum opto-electronic devices consist of hBN/MLG heterostructures, operate at accessible voltages, and can be assembled using readily-accessible fabrication techniques. We propose two distinct mechanisms for device operation versus bias polarity based on electrostatic charge switching of quantum emitters and drift of hot photoelectrons. Our results open a plethora of new opportunities in integrated quantum photonics with vdW materials. First, the ability to modulate and switch on/off quantum emitters is imperative for scalable quantum circuitry. Second, electrostatic gating can now be used to activate emitters post hBN growth and processing, and to select emitters at specific wavelengths. Third, a single device can now be employed to activate and tune quantum emitters into resonance to achieve indistinguishable photons from quantum emitters in hBN. Indeed, our results already show that under negative bias a nearly-coherent quantum source in hBN with linewidths of ~160 MHz can be obtained. Finally, and equally important, our results constitute the possibility to characterize charge transition levels of specific defects in hBN, and correlate them with theoretical studies of specific atomic defect structures. During the proof stage of the manuscript, we became aware of a complementary work on charge state control of defects in hBN [https://arxiv.org/abs/2202.09037].

## Materials and methods

### Preparation of hBN flakes

The hBN flakes are mechanically exfoliated onto 285 nm and 90 nm SiO_2_/Si substrates with Scotch tape from ultra high-purity bulk hBN crystals (carbon and oxygen impurity concentrations of <10^18^ cm^−3^). The hBN crystals are synthesized at high pressure and temperature of 4.5 GPa and 1500 °C respectively. The tape residuals on the flakes are removed through a 4-h calcination process in air at 500 °C using a hot plate.

### Plasma treatment for the hBN emitter generation

The plasma process is performed in a microwave plasma deposition system (SEKI AX5100). The exfoliated hBN flakes are placed on a graphite puck and then the chamber is pumped down to 1 × 10^–2^ Torr. After purging with argon for 10 min, 100 sccm H_2_ is induced and the chamber pressure is gradually increased to 60 Torr. The plasma power is set to 900 W, and hBN samples are treated for 3 min. Following this, a 40-min high-temperature (700 °C) annealing process is conducted in a tube furnace (Lindberg/Blue) in air, at a ramp heating rate of 120 °C min^−1^. The samples are cooled to room temperature naturally (normally takes 2–3 h to cool down to room temperature). After this, the UV ozone cleaning process is conducted in a UV ozone generator (ProCleaner™ Plus, Bioforce Nanosciences Inc.).

### hBN/MLG device fabrication

After the plasma treatment, the desired hBN flake on Si/SiO_2_ substrate is identified using a home-built scanning confocal PL microscope. The heterostructures are fabricated using an aligned transfer technique using polyvinyl alcohol (PVA) coated polydimethylsiloxane (PDMS) as a stamp. The gold electrodes of 5 nm Cr and 100 nm gold were fabricated using standard aligned photolithography and vacuum thermal deposition.

### Photoluminescence spectroscopy

The PL spectra were collected with a home-built scanning confocal microscope. The samples were excited with a 300 μW 532 nm continuous-wave (CW) laser. The laser reflection was spectrally filtered using a 532 nm dichroic mirror (LP03-532RE-25). Low-temperature optical measurements were performed using a similar confocal system with the sample mounted on the cold finger within an attoDRY800 cryostat (operating at 4 K). The emissions are collected with the spectrometer for spectra or two avalanche photodiodes (APDs) for photon counting. The second-order correlation (*g*^2^(τ)) measurements are conducted with a time-correlated single-photon counting module (Swabian, TimeTagger20) in a fiber-based Hanbury Brown-Twiss configuration with two APDs. Further information is available in the Supplementary Material.

### Theoretical calculations

Theoretical calculations reflect the linear drop of an electric field across a classical capacitance assuming all charge builds up at the contacts, i.e., *V*/*t*. To ensure the limited density of states of the non-metallic contacts were not significantly altering the classic capacitor behavior, a nonlinear Poisson equation was solved for both contacts assuming parabolic bands. Band diagrams reflect the band alignment from biasing the capacitance formed between a heavily doped p-type silicon (work function ɸ ~ 5 eV) and a 10 nm thick layer of graphite (ɸ ~ 4.6 eV). For simplicity a dielectric constant of 3.6 is used for both the hBN and SiO_2_, the electron affinity hBN is 2.3 eV and SiO_2_ is 0.9 eV^35,37^. Band bending effects of charge build-up in the non-metallic contacts were considered for both the graphite and Si contacts by numerically solving the classic one-dimensional Poisson equation with a Newton Raphson method provided by the diamond-banalyzer python package (https://pypi.org/project/diamond-bandalyzer/), although only resulted in ~0.2 eV shifts at 20 V, and thus were ignored.

## Supplementary information

Supplementary Information
