# Author Correction: Electrical control of quantum emitters in a Van der Waals heterostructure

**DOI:** 10.1038/s41377-022-00917-2

**Published:** 2022-07-18

**Authors:** Simon J. U. White, Tieshan Yang, Nikolai Dontschuk, Chi Li, Zai-Quan Xu, Mehran Kianinia, Alastair Stacey, Milos Toth, Igor Aharonovich

**Affiliations:** 1 School of Mathematical and Physical Sciences, University of Technology Sydney, Ultimo, NSW 2007 Australia; 2 ARC Centre of Excellence for Transformative Meta-Optical Systems, University of Technology Sydney, Ultimo, NSW 2007 Australia; 3 School of Physics, University of Melbourne, Parkville, VIC 3010 Australia; 4 School of Science, RMIT University, Melbourne, VIC 3001 Australia

**Keywords:** Optics and photonics, Optical materials and structures

Correction to: *Light: Science & Applications*

10.1038/s41377-022-00877-7 published online 20 June 2022

Correction

After publication of this article^1^, it was brought to our attention that the Figure 1 is incorrect, the correct Figure 1 is shown below:
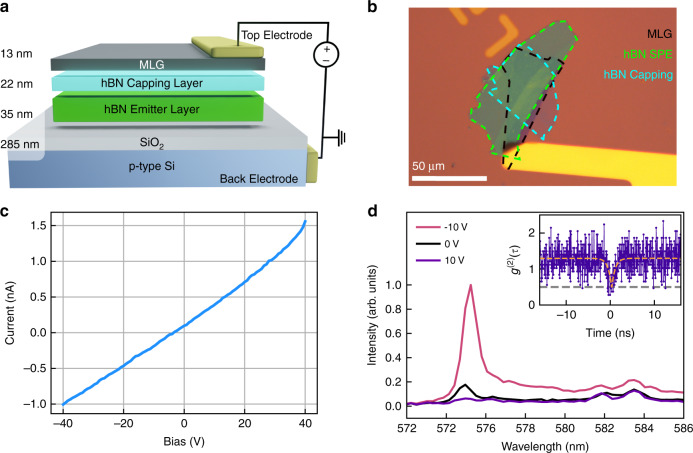


The original publication has been corrected.
