# Supplementary material for: Electrical control of quantum emitters in a Van der Waals heterostructure
Source: Light Sci Appl. 2022 Jun 20;11:186. doi: 10.1038/s41377-022-00877-7 (PMC9209426; doi:10.1038/s41377-022-00877-7)
Supplement: Supplementary file 1 — Supplementary Information [file 41377_2022_877_MOESM1_ESM.docx]

**Supplementary Information for:**

**Electrical Control of Quantum Emitters in a Van der Waals Heterostructure**

Simon J U White^1#^, Tieshan Yang^1,2#^, Nikolai Dontschuk^3^, Chi Li^1^, Zai-Quan Xu^1^, Mehran Kianinia^1,2^, Alastair Stacey^4^, Milos Toth^1,2^ and Igor Aharonovich^1,2^

1. School of Mathematical and Physical Sciences, University of Technology Sydney, Ultimo, New South Wales 2007, Australia

2. ARC Centre of Excellence for Transformative Meta-Optical Systems, University of Technology Sydney, Ultimo, New South Wales 2007, Australia

3. School of Physics, University of Melbourne, Parkville, Victoria 3010, Australia

4. School of Science, RMIT University, Melbourne, Victoria 3001, Australia

# These authors contributed equally

**The supporting information includes:**

- *hBN/MLG Heterostructure Device Fabrication*
- *Confocal Photoluminescence Schematic*
- *Device Dimensions and AFM*
- *Device Electrical Characteristics*
- *Room Temperature Spectral Characterization*
- *Cryogenic Characterization*
- *Second-order Correlation Analysis*
- *Photoluminescence Activation and Switching Rates*

**hBN/MLG Heterostructure Device Fabrication**

After the plasma treatment of hBN flake to activate single photon emitters, the desired hBN flake on SiO_2_/Si substrate is identified using optical microscopy. To pick up and transfer the hBN/MLG (multilayer graphene) flakes, a dry transfer method with a home-built transfer platform is used. We use a stamp consisting of a thin film of polyvinyl alcohol (PVA) mounted on a block of polydimethylsiloxane (PDMS) for mechanical support. The stamp is placed on a glass slide attached to a micromanipulator under a microscope. The SiO_2_/Si substrate with MLG or hBN capping flakes to be transferred, are fixed onto a transfer stage, and positioned with a micromanipulator.

Firstly, the PVA stamp is placed into contact with a selected MLG flake. Next, we lift the PVA stamp with MLG flakes from the SiO_2_/Si substrate at room temperature.

Then, we remove the SiO_2_/Si substrate and place the SiO_2_/Si substrate with hBN capping layer onto the transfer stage. We position the MLG flakes on a PVA stamp over a selected region of the target SiO_2_/Si substrate with hBN capping layer and bring the PVA stamp and target SiO_2_/Si substrate with hBN fully into contact. Next, we lift the PVA stamp with MLG/hBN flakes from the SiO_2_/Si substrate at room temperature.

Next, we remove the SiO_2_/Si substrate and place the SiO_2_/Si substrate with hBN emitter layer onto the stage. We position the hBN/MLG flakes on a PVA stamp over a selected region of the target SiO_2_/Si substrate with hBN emitter layer and bring the PVA stamp and target substrate with hBN emitter layer fully into contact. Then, the stage was heated up and reached about ~60 ℃. After this, we lift the glass slide with a PDMS block. The PVA stamp remains on the target substrate due to the preferential adhesion. Finally, we use water to dissolve the PVA stamp and clean the hBN/MLG heterostructure device surface.

Then, the electrode pattern is fabricated by a photolithography method on top of the MLG layer followed by a thermal deposited 5 nm/100 nm thick Cr/Au electrode pad (a gold pad with dimension ~ 600 × 600 µm^2^). Finally, we use acetone to lift off the non-patterned Cr/Au area and use acetone and isopropyl alcohol (IPA) to clean the hBN/MLG heterostructure device surface.

**Confocal Photoluminescence Schematic**

A continuous wave (CW) 532 nm laser (Gem 532™, Laser Quantum Ltd.) is used for excitation and photoluminescence (PL) scanning. The beam is directed via a 532 nm dichroic mirror (LP03-532RE-25) into the attoDRY800 cryostat and focused to a diffraction-limited spot (~ 600 nm) on the sample using a vacuum compatible high numerical aperture objective lens (attocube LT-APO/VISIR 0.82 NA). A 4*f* lens system and X-Y piezo scanning mirror (FSM-300™) is used to scan the spot over the sample. The collected light is filtered using the dichroic mirror and an additional long-pass filter (568 LP filter, Semrock™). Confocality is achieved using a 62.5 graded index multimode fibre as a pinhole in collection. The signal is then collected using this fiber and directed to a spectrometer for spectral characterization (room temp measurements using a Princeton Instruments Acton SpectraPro™ with a 1200 lines mm^-1^ grating, and cryogenic measurements using an Andor Shamrock 303i with 300 and 1800 lines mm^-1^ gratings). The multimode fiber can also direct the PL to avalanche photodiodes (Excelitas SPCM-AQRH) for single-photon counting, PL mapping, and second-order correlation measurements. Second-order correlation measurements are made using a fiber-based Hanbury-Brown and Twiss setup and a time-correlated single photon counting module (Swabian, TimeTagger20). A bias is applied to the device (within the cryostat via vacuum feedthrough) using either a Keithley Tektronix sourcemeter (2612B) SMU or an Agilent function generator (33522A). A similar optical path is used when exciting the sample with a tunable dye laser (Sirah Matisse 2 DX), which can be tuned between 570 nm and 610 nm. In this case, the dichroic mirror is replaced with a 90/10 (transmission/reflection ratio), and the reflected laser is rejected using a Semrock long-pass filter.

**
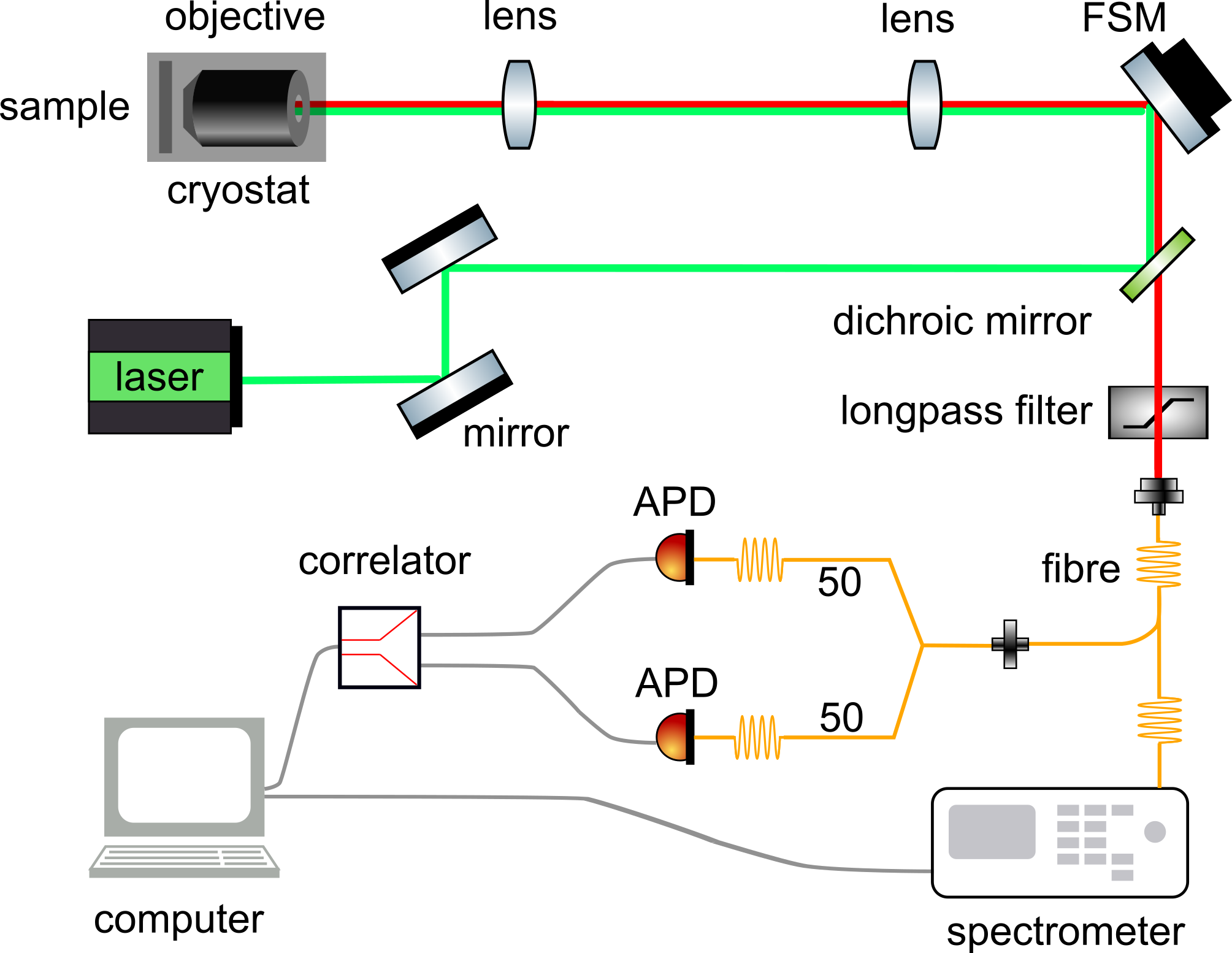
**

***Figure S1****. Schematic of the confocal photoluminescence setup (FSM, fast steering mirror; APD, avalanche photodiodes). The dichroic mirror is replaced by a 90/10 beam splitter for excitation using a tunable laser.*

**Device Dimensions and AFM**

An optical microscope image and AFM of the hBN/MLG heterostructure device on the 90 nm SiO_2_/Si substrate is shown in Figure S2. A hBN capping layer is introduced to prevent emitters in the hBN layer from quenching during the device fabrication. Non-contact mode AFM was used to create the image shown in Figure S2b, it displays the target flake area. The pink line at the top of the image marks the location used to measure the thickness of the hBN SPE layer, which shows the thickness of ~35 nm (Figure S2c). The red line at the top of the image marks the location used to measure the thickness of the MLG, which shows the thickness of ~13 nm (Figure S2d). The blue line at the bottom of the image marks the location used to measure the thickness of the hBN capping layer, which shows the thickness of ~22 nm (Figure S2e).


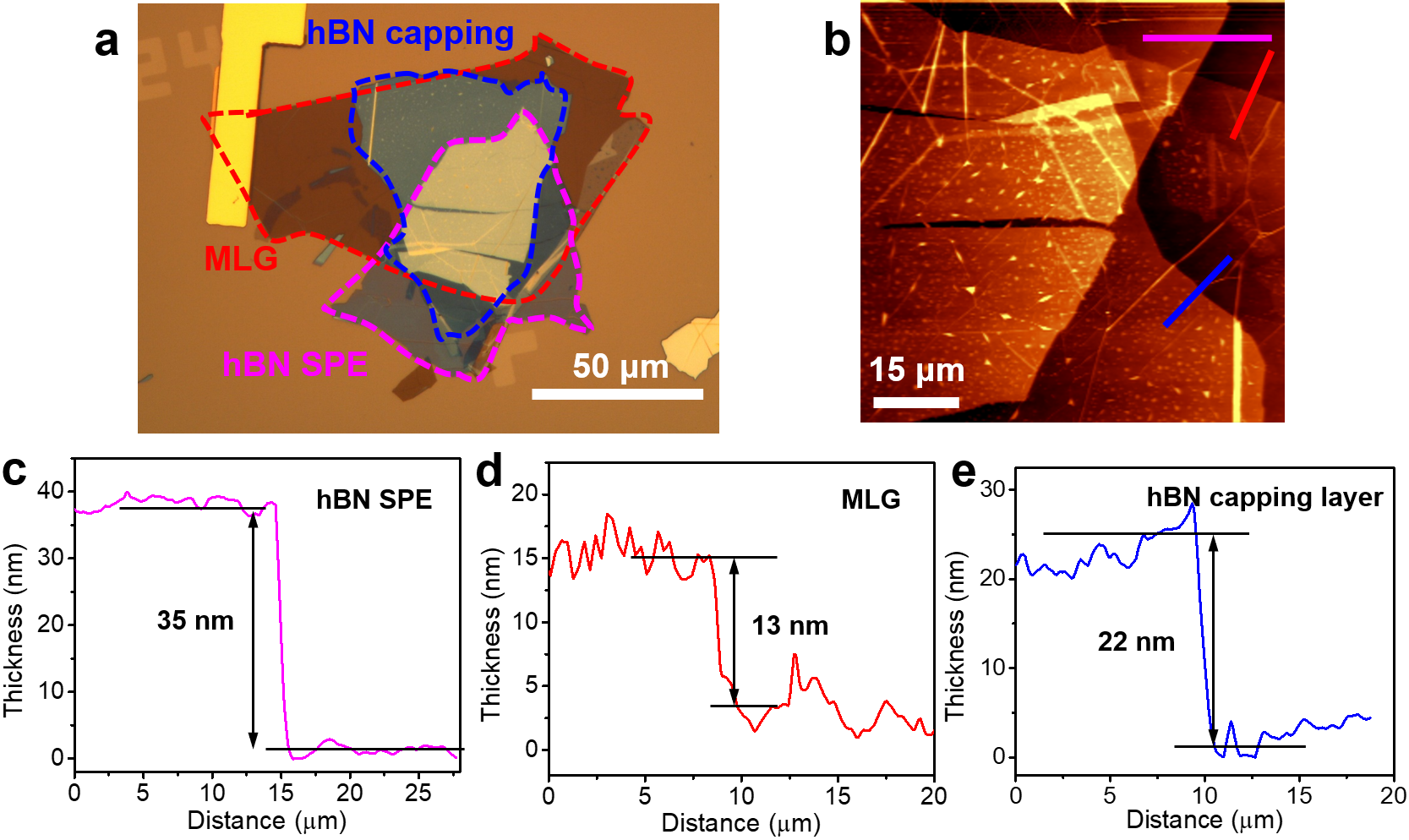


***Figure S2****. hBN/MLG heterostructure device.* ***a, b.*** *Optical microscope image (a) and AFM image (b) of the devices on a p-type silicon with a 90 nm thick thermal oxide layer. Optical image of a device based on MLG/hBN(capping)/hBN(emitters) placed on p-type silicon with a 90 nm thick thermal oxide layer. Each layer is outlined in dash lines.* ***c-e.*** *The height outline of the hBN emitter layer (c), graphene layer (d), and hBN capping layer (c). The thickness of the hBN emitter, MLG, and hBN capping layers are 35 nm, 13 nm, and 22 nm respectively.*

**Device Electrical Characteristics**

To study the current vs. voltage characteristics of the hBN/MLG heterostructure device with 285 nm thermal silicon oxide layer, we apply a bias between the gold electrode on the graphene and the p-type Si and measure the instantaneous current. We perform voltage sweeps from -20 V to +20 V and back to -20 V with differing step times (*Δt*) and 0.4 V steps. As seen in Supplementary Figures 3a and 3b, there is no significant photocurrent in the hBN/MLG heterostructure. The leakage current is also negligible under short time scales (voltage step time *Δt* = 0.001 s, leakage current < 5 nA), and this reduces further over time. Interestingly, the device also shows a capacitive behavior when the bias is applied on time scales less than 0.1 s. We note here, the charging behavior cannot be isolated to only the hBN/MLG interface and is likely also contributed to by the Si/electrode, MLG electrode interfaces, and also the SiO_2_/electrode interface, as the electrode is not confined only to the graphene as seen in Figure 1b of the main.

*
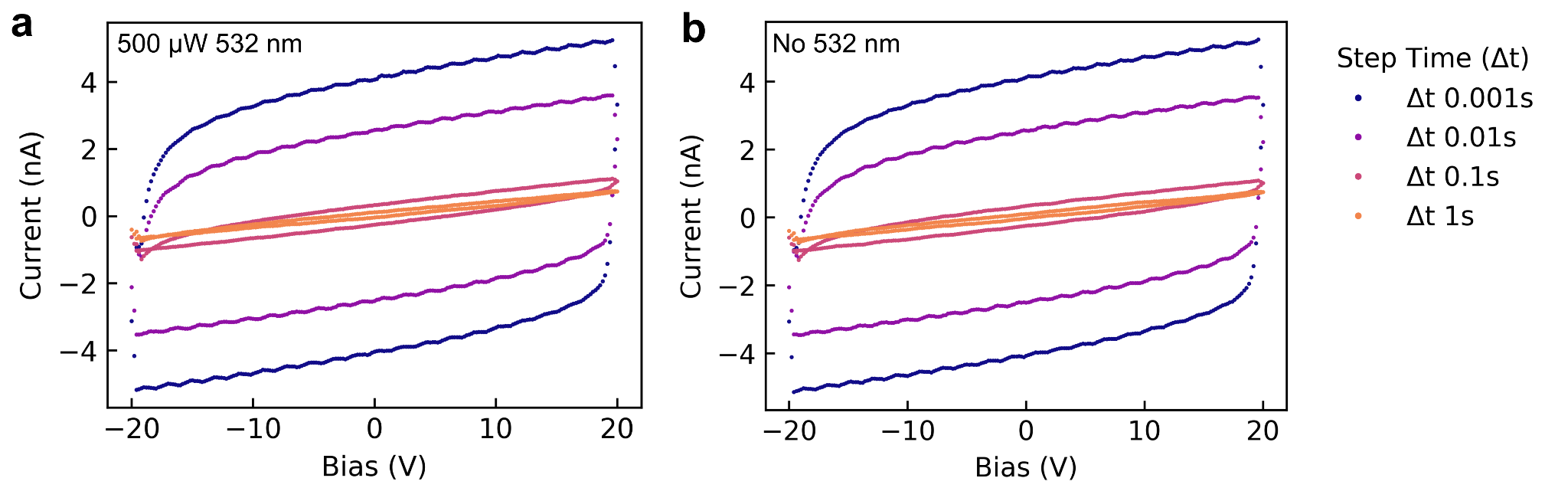
*

***Figure S3****. I-V characteristics of hBN/MLG heterostructure with 285 nm thermal silicon oxide layer.* ***a.*** *Current vs. voltage curves for voltage step time of 0.001 to 1s with 532 nm laser excitation on the heterostructure.* ***b.*** *Current vs. voltage curves for voltage step time of 0.001 to 1s without 532 nm laser excitation (dark) on the heterostructure.*

The I-V characteristics of a hBN/MLG heterostructure device with a 90 nm thermal silicon oxide layer is also shown in Figure S4. Here the step time (*Δt*) is kept at 1s and the leakage current is maintained below 100 nA for a bias range between -2 V and +1 V.

*
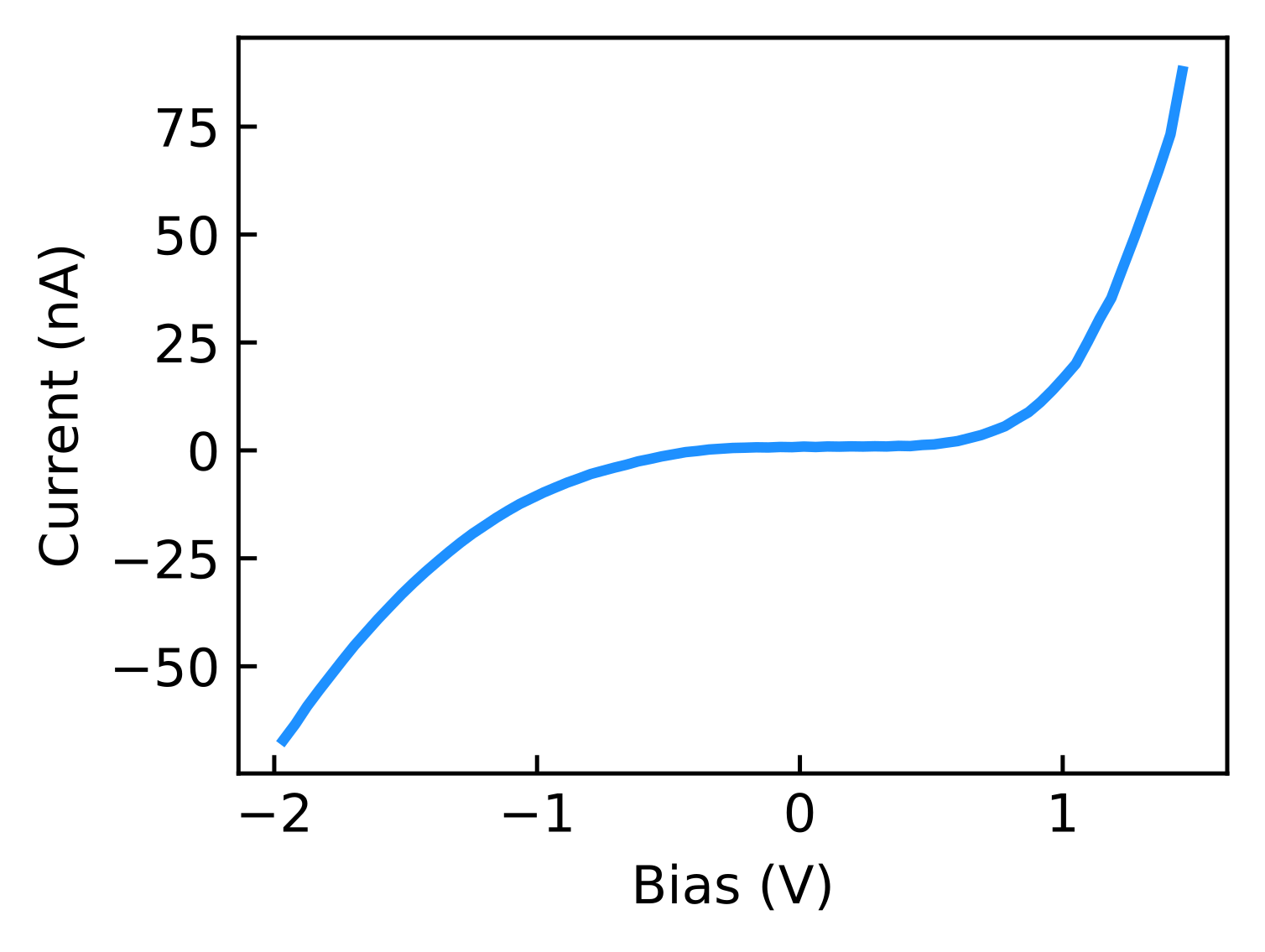
*

***Figure S4****. I-V characteristics of hBN/MLG heterostructure with 90 nm thermal silicon oxide layer.*

**Room Temperature Spectral Characterization**

We also study the optical properties of the emitters in the hBN/MLG heterostructure device at room temperature. To provide a broader picture of the photophysical properties of emitters under applied bias, we record the behavior of a large ensemble of emitters. A series of spectra recorded at different biases from -10 V to 10 V is shown in Figure S5, and a photoluminescence peak is observed to respond to the applied bias. The emission at a wavelength of 666 nm is completely dark in the absence of bias but begins to appear around 2.5 V, reaching its maximum at 6 V, and appears very dim under a negative bias from 0 V to -10 V.

*
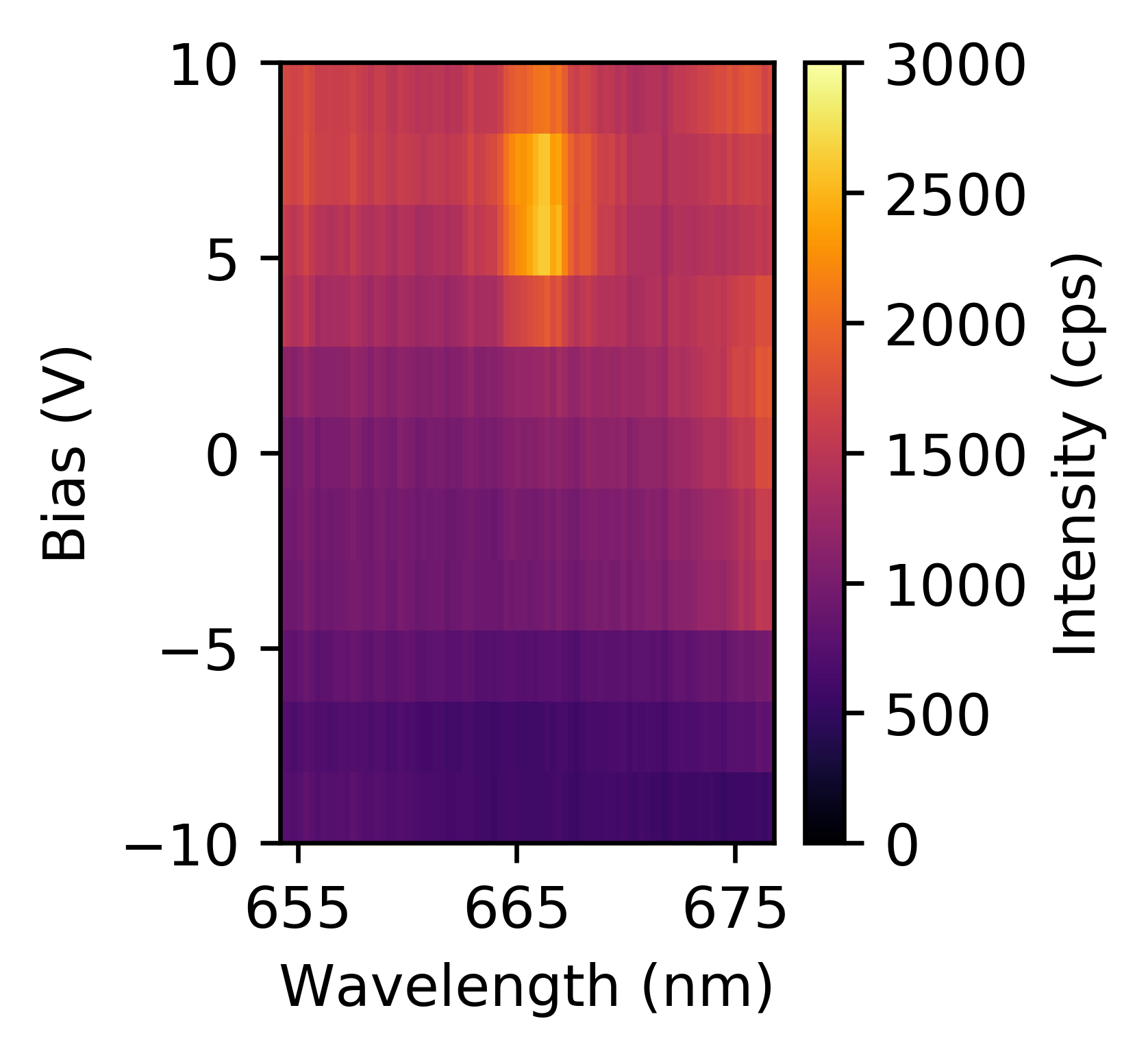
*

***Figure S5****. Room-temperature PL spectra recorded over a bias range of -10 V to 10 V displaying activation of a 666 nm emission peak at 7 V.*

**Cryogenic Spectral Characterization**

Electrical control of the hBN quantum emitters in the hBN/MLG heterostructure device on 285 nm SiO_2_/Si substrate is shown in Figure S6. The PL spectra under different bias from two emitters are plotted in Figure S6a and S6b. In order to clarify, we normalized the spectra intensity. As shown in Figure S6a, the emitter is activated at ~8 V and its intensity continues to increase and then the emitter is switched off at ~22 V. While Figure S6b demonstrates a different phenomenon. The emitter's intensity increases monotonically as the bias drops from 0 V to -30 V.


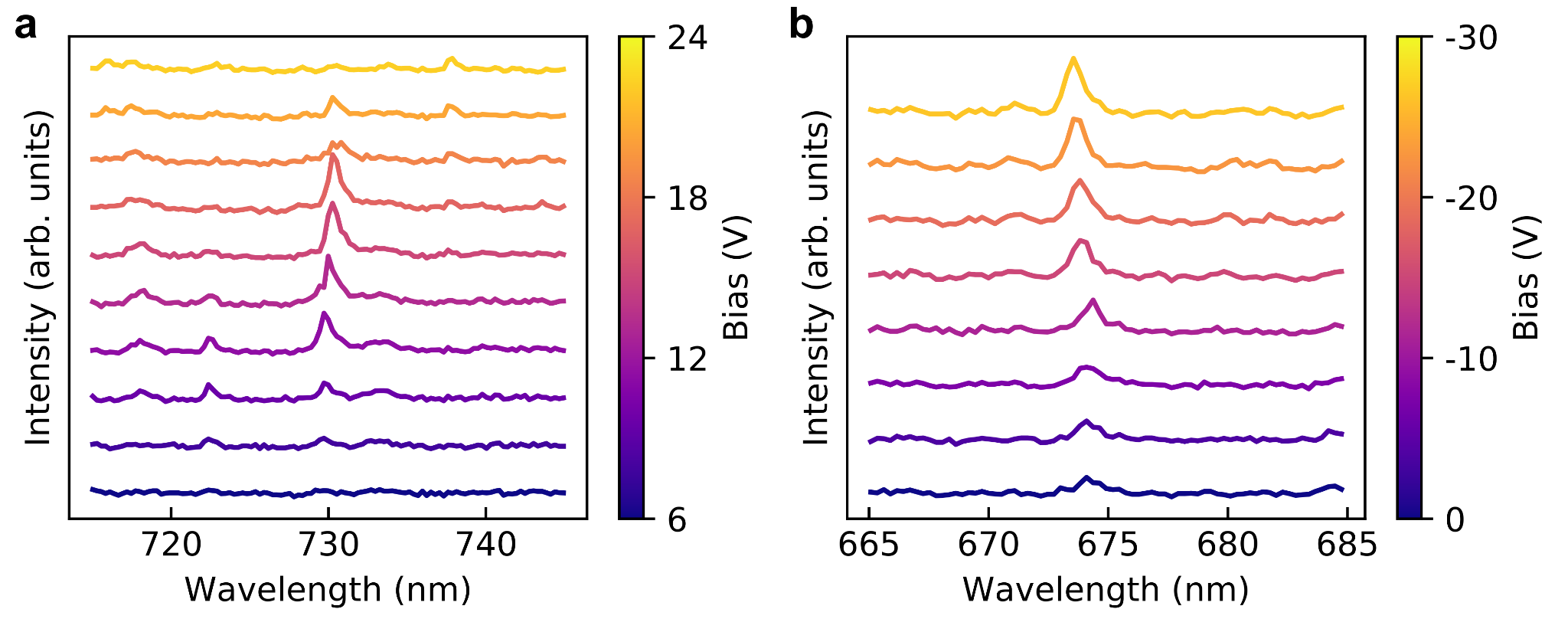


***Figure S6****.* ***a, b.*** *Normalized PL spectra of two different emitters at a positive bias of 6 V to 24 V (a) and negative bias of 0 V to -30 V (b), respectively.*

To further corroborate our findings, we provide PL spectra *vs.* bias scans for various emitter regions on the hBN/MLG heterostructure device with 285 nm silicon oxide spacer. Figure S7 displays hBN emitter activation across the complete visible spectrum from 580 to 800 nm. A bright emission band under +20 V bias is confirmed at various positions across the hBN flake, as illustrated in Supplementary Figures 7a and 7b. The distribution of hBN charge state depths is clearly observed in 7a as individual emitters observe differing optimal +ve and -ve biases. The majority of emitter PL peaks show minimal shift in energy under an applied bias, as the Stark shift depends principally on the orientation of the bias and emitter dipole. The majority of hBN single photon emitter dipoles are thought to lie in-plane and no Stark shift is observed. For a few emitters, as highlighted in Figure S7c, the dipole moment lies out of plane, possibly due to wrinkles and/or grain boundaries of the hBN flake. Here, the applied field vector would be parallel to the dipole moment thus a shifting of PL energy can be observed.

*
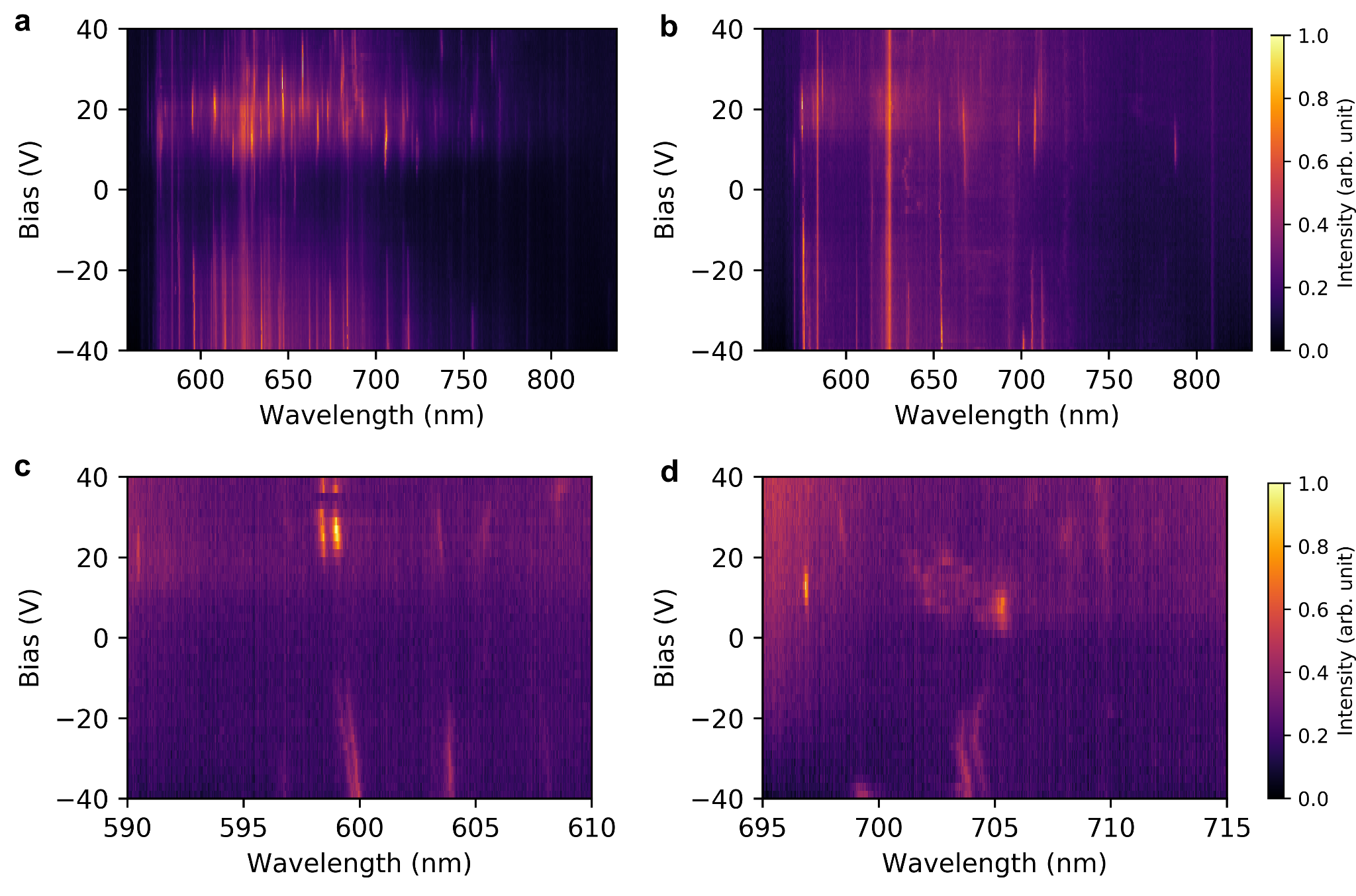
*

***Figure S7****. Further modulation and Stark shift of hBN quantum emitters in the heterostructure device with 285 nm silicon oxide.* ***a, b.*** *PL spectra recorded as a function of bias, over the range of -40 to +40V for various regions of hBN emitters.* ***c.*** *hBN emitter PL spectra with DC Stark shifts and variable activation bias.* ***d.*** *Individual hBN activated specifically under +ve or -ve bias.*

Electrical control of the hBN quantum emitters in a hBN/MLG heterostructure device on a 90 nm thermal oxide layer is also shown in Figure S8. We observe similar behavior to the device with 285 nm oxide layer with smaller magnitudes of bias, as expected for an increased effective field proportional to the device thickness. As seen in the main, a band of increased PL is observed for a range of emitters, this time at a bias of +4 V, with subtle changes in optimal bias from emitter to emitter. Also, similarly a dark band is observed for the majority of emitters when both electrodes are grounded, with fewer emitters activated in the region. Finally, we also observe the monotonic increase in PL under a negative bias.

*
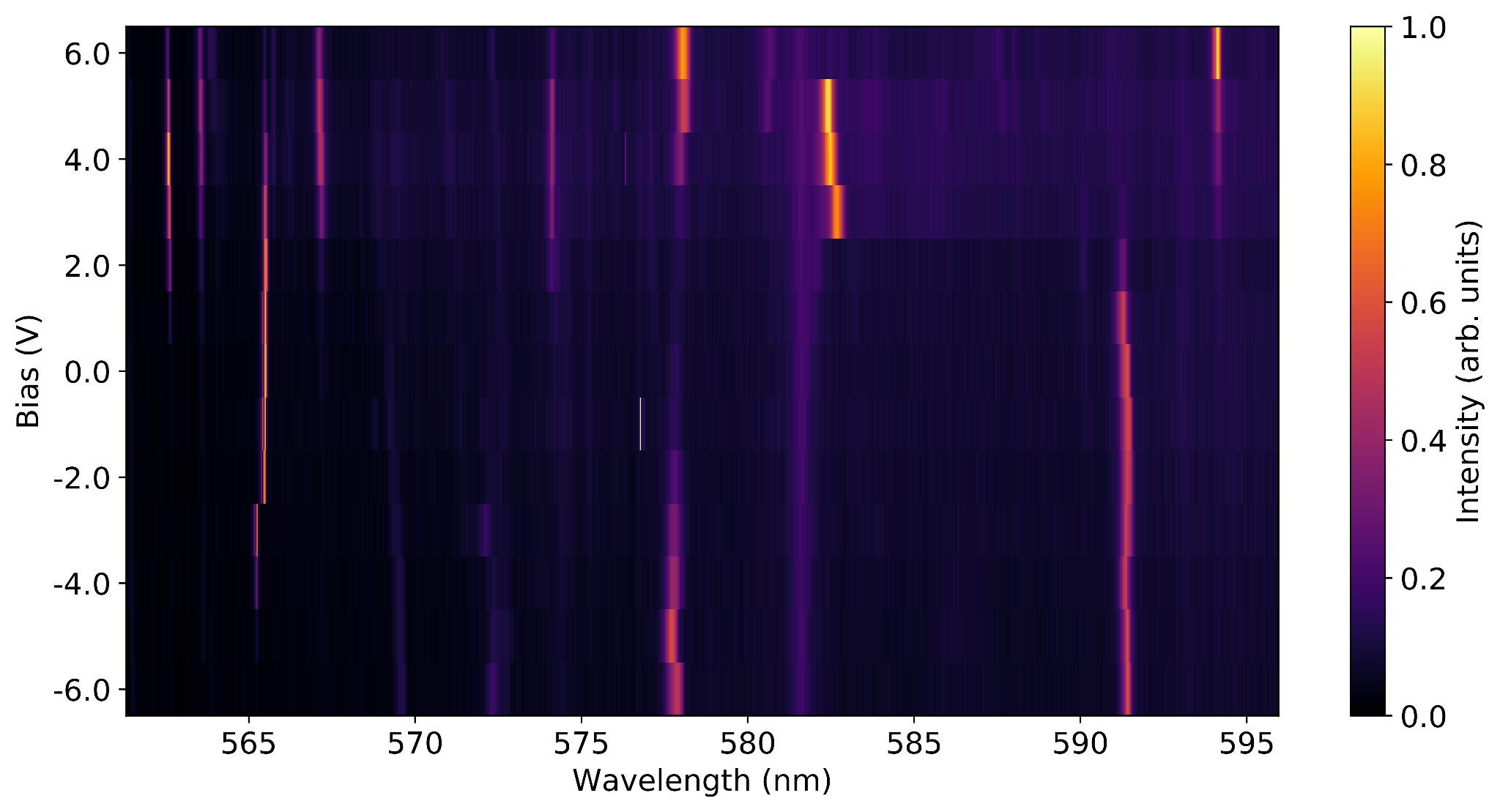
*

***Figure S8****. Series of PL spectra from hBN emitters at biases ranging from -6 V to 6 V for the heterostructure device with 90 nm silicon oxide.*

Finally, the bias-dependent PL spectra from two emitters are plotted in Figure S9. The spectra are normalized for clarity. Two different behaviors, under positive and negative bias, are observed. The enhanced PL of an emitter under positive bias is seen in Figure S9a, with an activation bias around -0.5 V. As the bias is increased to 3V the PL increases substantially. Individual spectra for the same emitter are plotted for -2 V, 0 V and 3 V bias to demonstrate the significant PL increase, as seen in Figure S9c. A similar case is also shown for a different emitter under negative bias in Figure S9b. In this case the emitter is active but relatively dim at 0 V bias. As the bias is dropped to -2 V the PL intensity increases, with individual spectra plotted in Figure S9d.

***
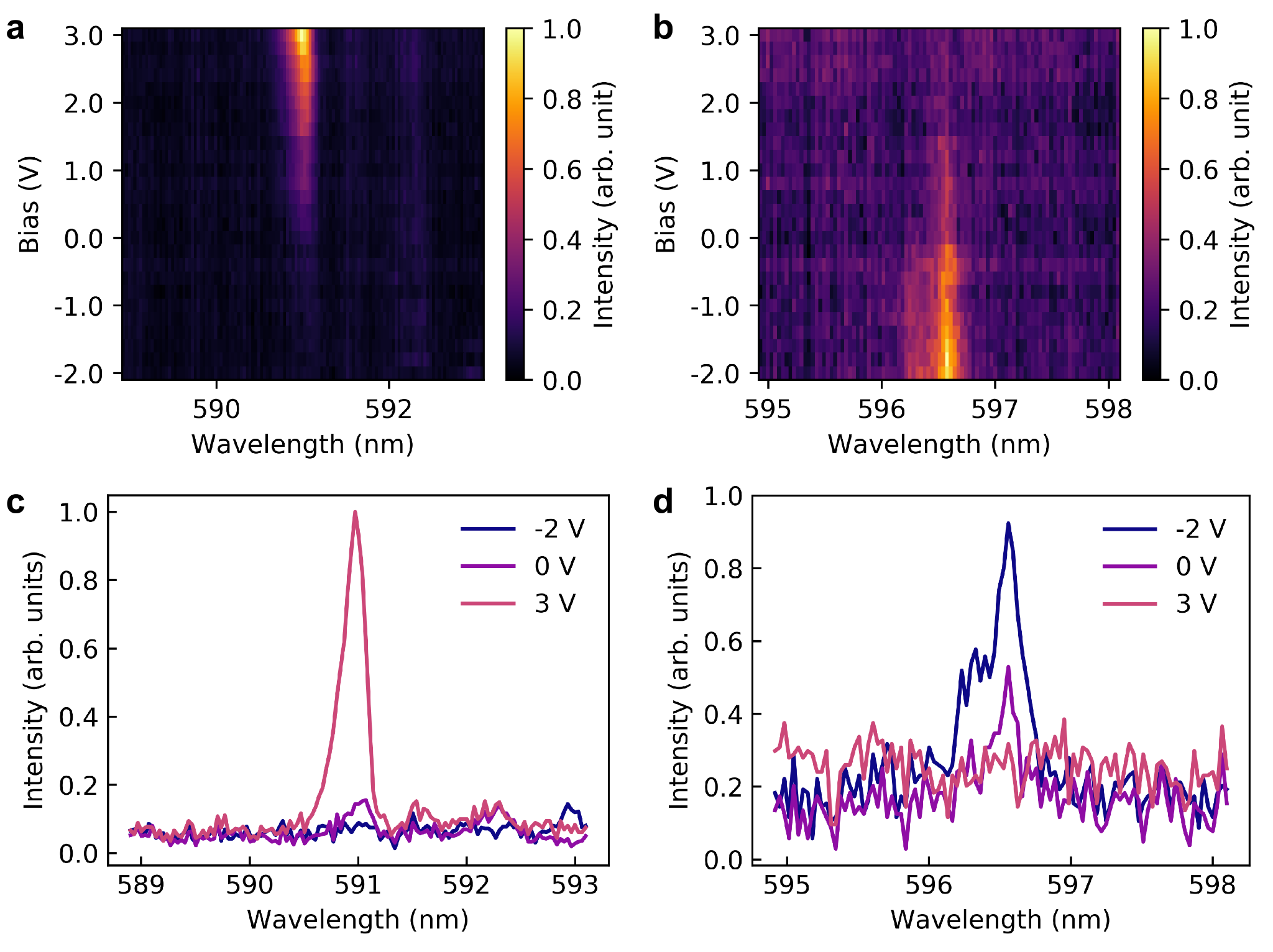
***

***Figure S9.*** *Controlled switching of hBN emitters in the quantum heterostructure device.* ***a, b.*** *Series of normalized PL spectra recorded two different emitters at a bias range of -2 V to 3 V.* ***c, d****. Normalized PL spectra of two different emitters at a bias of -2 V, 0 V, and 3 V, respectively. Excitation laser power: 200 μW.*

Figure S10 shows a typical intensity trace to emphasize the optical stability of the studied quantum emitters at 4 K with no blinking over the course of one minute.

*
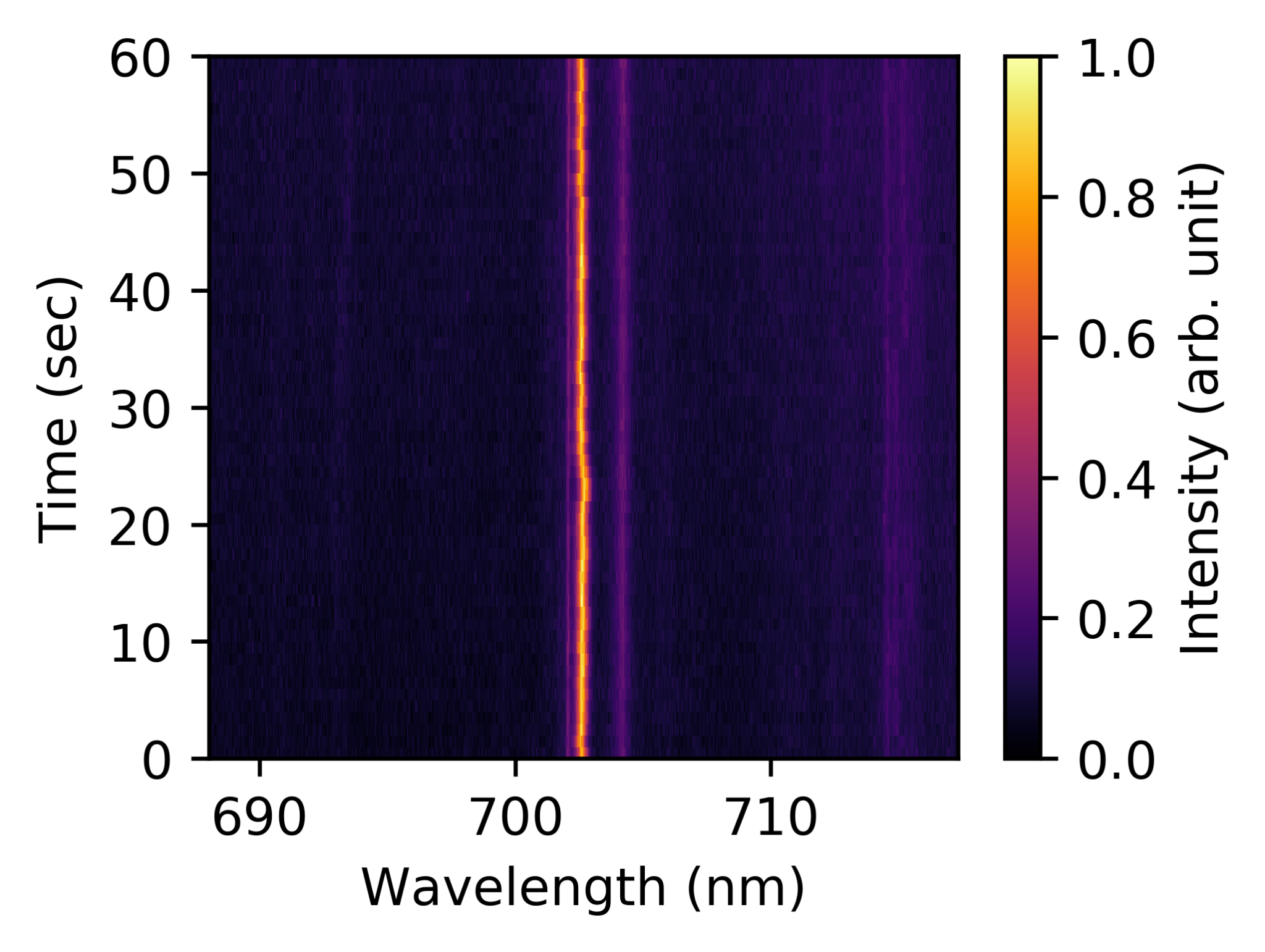
*

***Figure S10****. PL time series from the same emitter at a 1 s time interval for a 60 s period with 0 V bias at 4 K. The intensity and the peak position remain identical throughout the whole process, suggesting this emitter has no blinking on this time scale.*

**Second-order Correlation Analysis**

Second-order correlation measurements were performed on a number of photoluminescence lines using a fiber based Hanbury Brown-Twiss (HBT) intensity interferometer, and tunable band pass filters to isolate a single photoluminescence peak. The data is normalized in real time using the count rates on each detector as given by the equation below^1^.

$$g^{\left( 2 \right)}\left( t \right)=\frac{C\left( t \right)}{N_{1}N_{2}⍵T}$$

Where *C(t)* is the un-normalized correlations, *N_1_* and *N_2_* are the detector count rates, *⍵* is the bin width and *T* is the integration time.

The second-order correlation data were fitted with the three-level model.

$$g^{\left( 2 \right)}\left( t \right)=1-\left( \left( 1-A \right)+A_{m} \right)e^{\frac{-t}{\tau}}+A_{m}e^{\frac{-t}{\tau_{m}}}$$

Where *A* is equal to antibunching minimum (g^(2)^(0)), *A_m_* is the bunching amplitude associated with the metastable state, and *τ* and *τ_m_* are the lifetimes for the excited state and the metastable states respectively^2-4^.

Further analysis of the emitter shown in Figure 1d is shown in Figure S11. A narrow PL peak is observed at 575 nm, which responds strongly to the influence of an applied bias. Under a +10 V bias, the PL is completely quenched, whereas under a -10 V bias, the PL increases significantly. To determine whether the PL is from a single emitter, second-order correlation data is recorded using a fiber based HBT interferometer, and a tunable filter to create a bandpass of 575 ± 2 nm. Long time scale correlations (shown in Figure S11d) reveal bunching from the emitter (A_m_ = 0.3, τ_m_ = 1.3 μs) due to an additional metastable state. Short time scale correlations reveal a dip with g^(2)^(0) = 0.48 ± 0.12, and an excited state lifetime of τ = 0.77 ± 0.16 ns, as shown in Figure S11c). Measurements are not background corrected as the background PL changes as a function of bias due to the high density of emission lines. This results in the fit g^(2)^(t=0) representing an upper bound for the emitter.

**
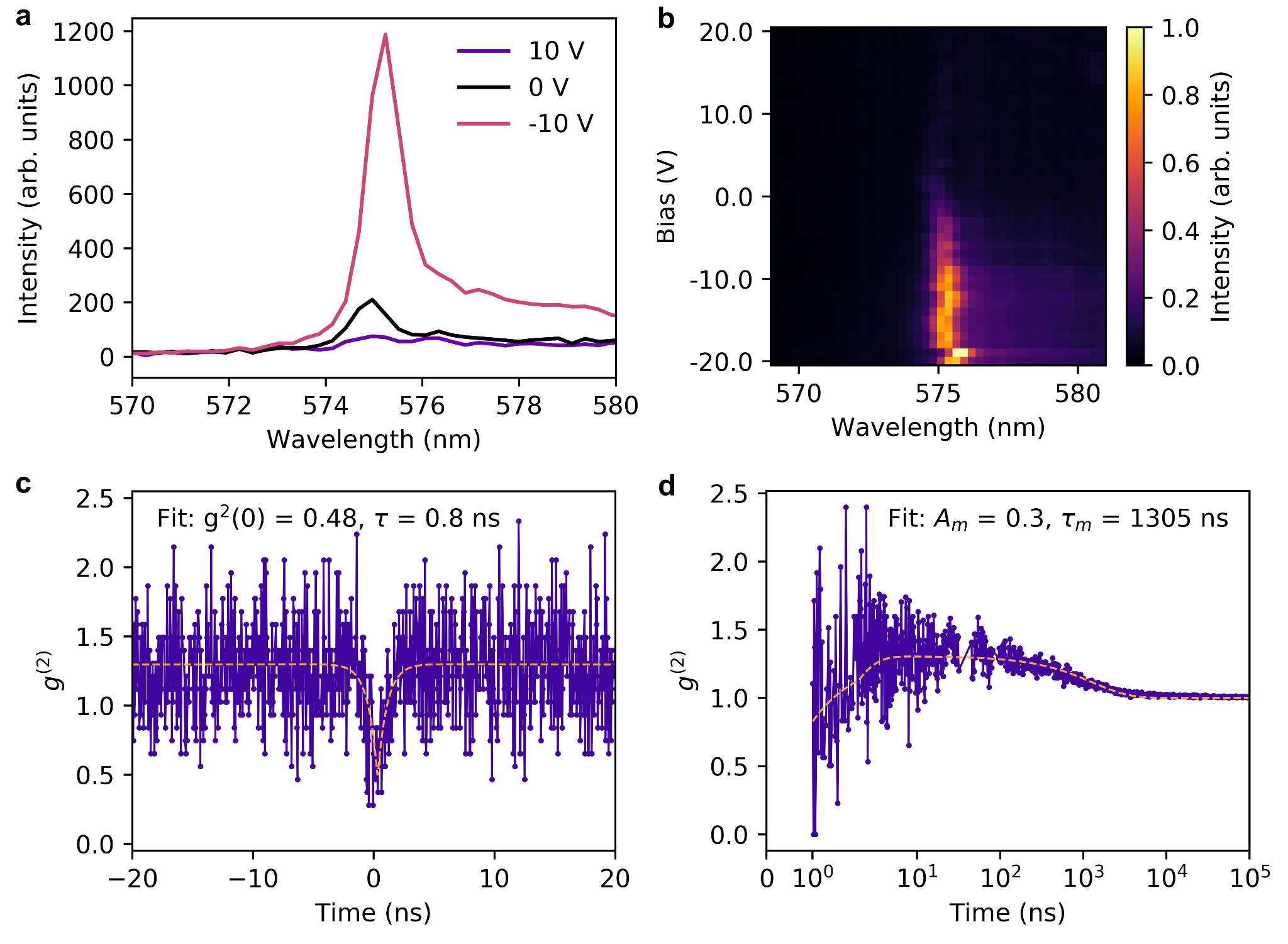
**

***Figure S11****. Emitter PL and second-order correlation for an emitter activated under a bias.* ***a.*** *Cryogenic PL spectra of an emitter in the heterostructure under a bias of -10 V (red), 0 V (black) and 10 V (purple), with 300 μW 532 nm excitation.* ***b.*** *Series of normalized PL spectra recorded over a bias range of -20 V to 20 V.* ***c.*** *Short time scale second-order correlation data measured from the same emitter under -10 V bias (purple). The PL was filtered using a tunable bandpass filter centered at 575 ± 2 nm and the fit (dashed yellow) reveals g^(2)^(0) = 0.48, without background correction.* ***d.*** *Long time scale second-order correlation data between 1 ns and 0.1 ms revealing bunching due to an additional metastable state.*

Background correction was unviable for these emitters as is shown below in Figure S12. Here the conventional process is used where count rates are recorded while the emitter is fluorescing and while it is not (Figure S12a). The mean count rate over 10 s is recorded and used to estimate the signal to noise ratio for the emitter. This signal to noise ratio is then applied the normalized second-order correlation data by the equation below^1^.

$$g^{\left( 2 \right)}\left( t \right)=\frac{\left[ g_{N}^{\left( 2 \right)}\left( t \right) -\left( 1-\rho^{2} \right) \right]}{\rho^{2}}$$

Where *⍴* = signal/(signal + background) and the non-background corrected normalized second-order correlations is g_N_^(2)^. As seen in Figures S12b and S12c, corrected second-order correlation dip is not below 0.5, indicating either multiple single emitters or a single emitter with significant background PL.

*
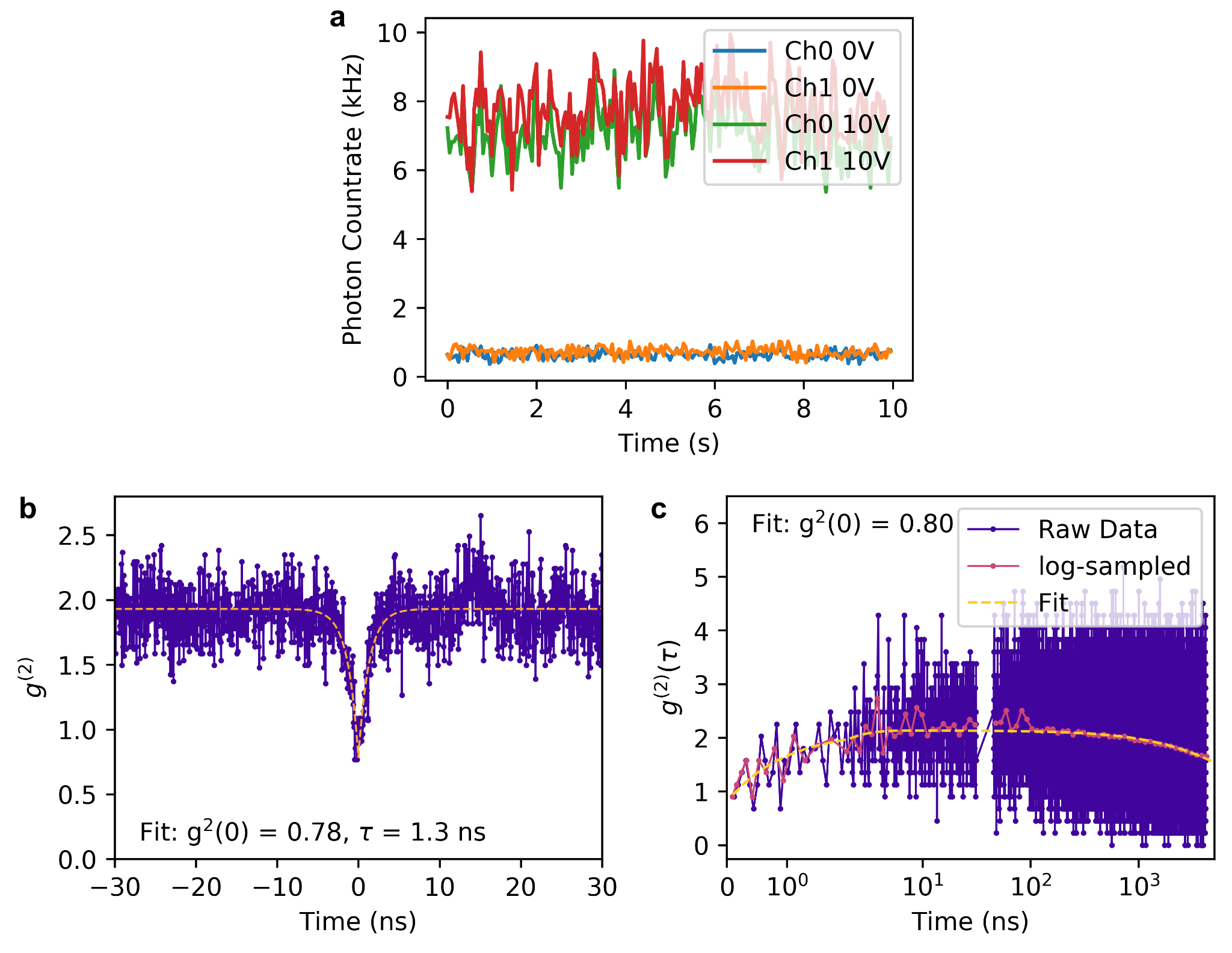
*

***Figure S12****. Second-order correlation and background correction for an emitter activated under a bias.* ***a.*** *Cryogenic PL time trace measurements recorded on each detector are shown with and without a 10 V bias.* ***b.*** *Short time scale second-order correlation displaying a dip at 0 delay time. The fit reveals a g^(2)^(0) = 0.78 and an excited state lifetime of 1.3 ns.* ***c.*** *Long time scale second-order correlation reveals bunching that lasts over 1000 ns, likely due to an additional metastable state. Measurements were made using 300 μW 532 nm excitation.*

To demonstrate the nonclassical nature of emissions across the entire observed spectrum we filter a 762.5 nm emission peak using a narrow ± 2 nm band pass filter. The peak is activated under a +14 V bias as shown in Figure S13. Second-order correlation measurements reveal a dip at 0 delay time and significant bunching due to the presence of an additional metastable state. The fit reveals a dip of g^(2)^(0) = 0.51 and an excited state lifetime of 2.1 ns.


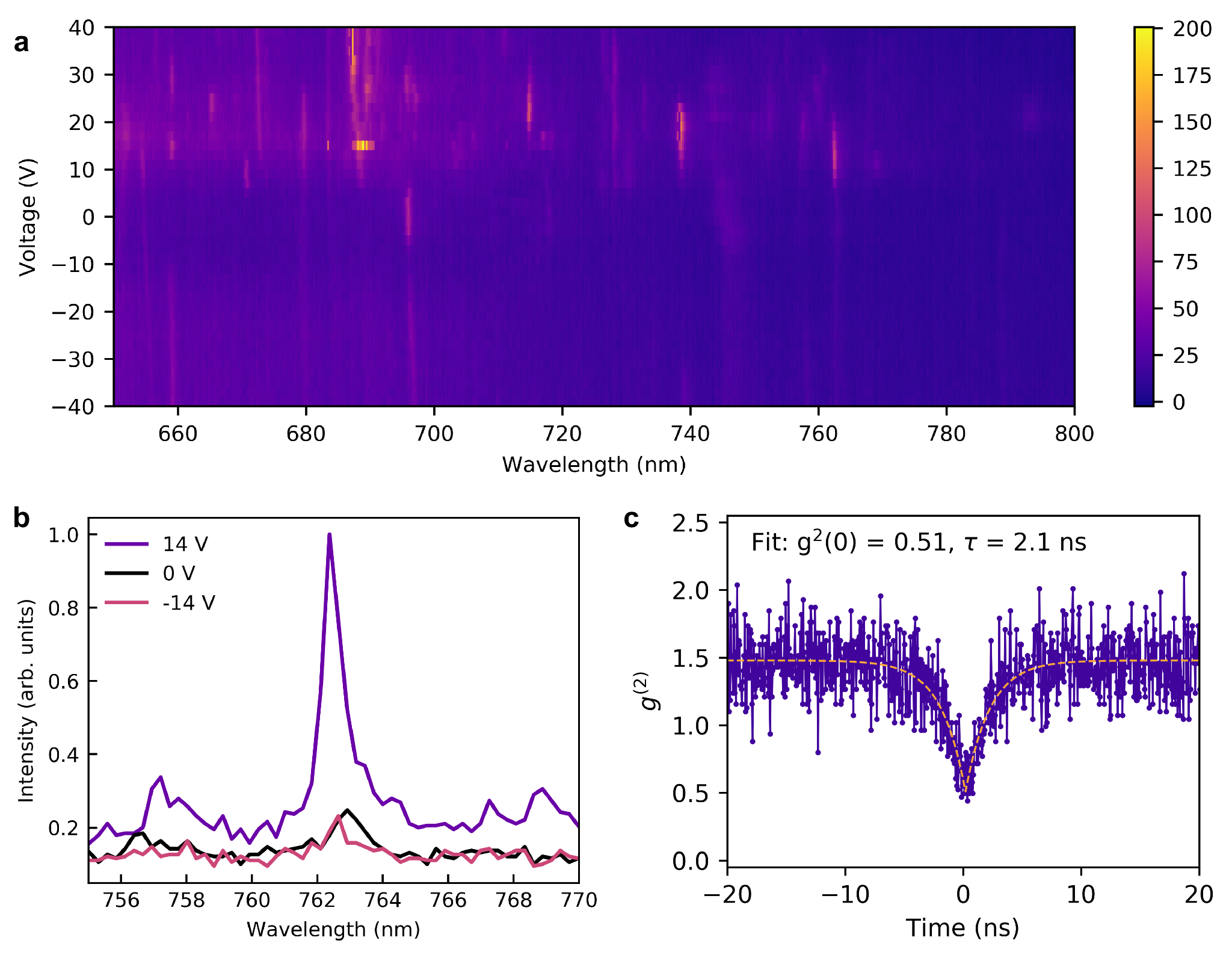


***Figure S13****. Second-order correlation for an emitter at 760 nm.* ***a.*** *PL spectra versus bias displaying multiple emission peaks.* ***b.*** *A single isolated PL peak centered at 762.5 nm activated a +14 V.* ***c.*** *Normalized second-order correlation data of the 762.5 nm peak filtered using a narrow ± 2 nm band pass filter. The data displays a dip at 0 delay time revealing nonclassical nature of the emission and the fit reveals a dip of g^(2)^(0) = 0.51 and an excited state lifetime of 2.1 ns. Measurements were made using 300 μW 532 nm excitation.*

**PL Activation and Switching Rates**

The PL behavior for an emitter under an applied bias is shown in Figure S14. The sample was excited with a 300 μw 532 nm laser at 5 K. The PL peak observed at 593 nm is shown to increase in intensity under both positive and negative bias, and a small stark shift, ~ 0.5 nm, is also observed over a ± 20 V sweep. The intensity modulation is observed to be repeatable as shown in Figures S14b and S14c. The activation time under positive and negative bias was measured and is displayed in Figures S14d and S14e. The fit of a single exponential reveals τ_on_​ = 43 ± 0.8 ms and 8.3 ± 0.3 μs, for +10 and -10 V respectively. This vast difference in activation time (2.5 orders of magnitude) supports the differing activation mechanisms described in the main text.

*
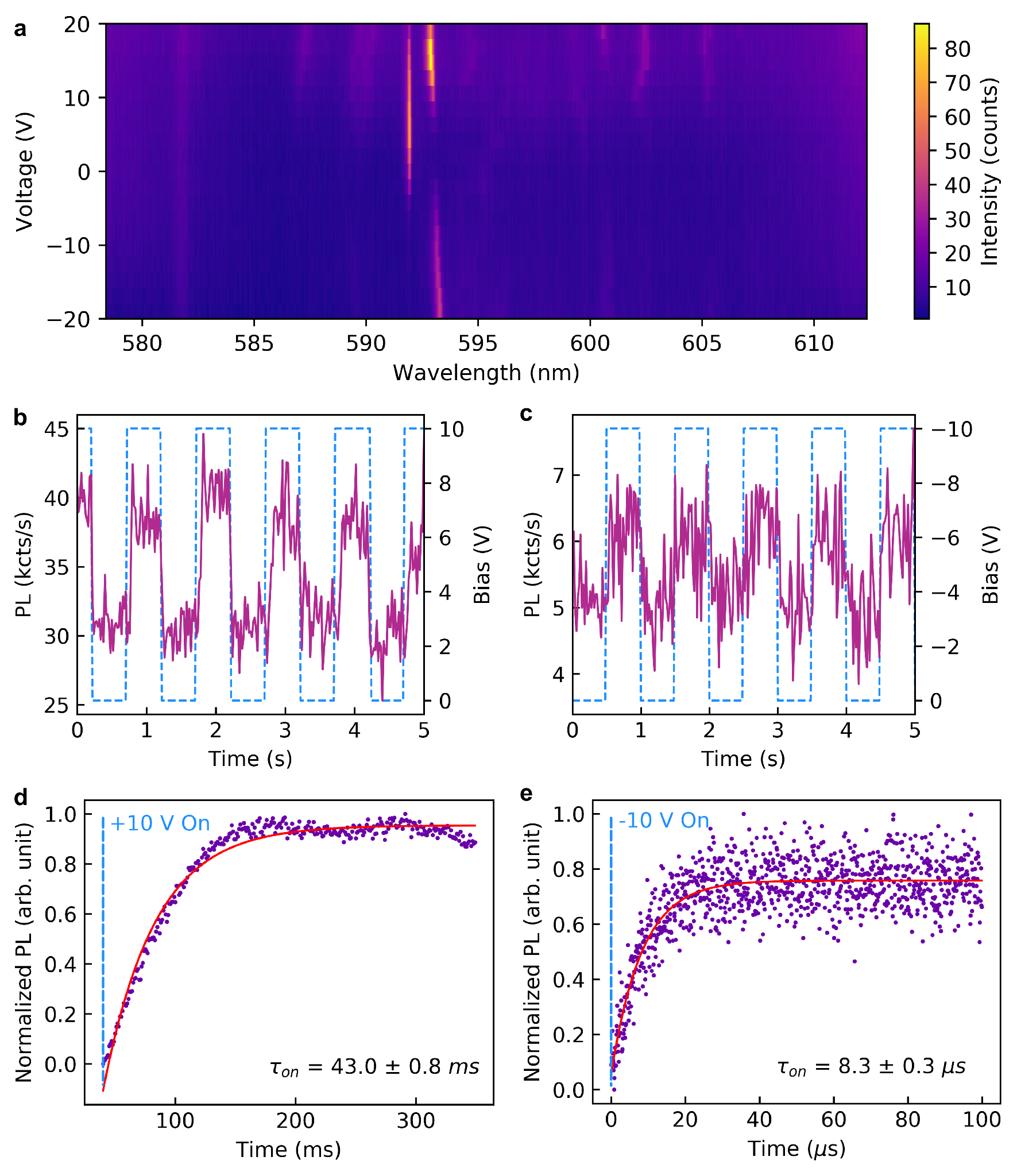
*

***Figure S14****. PL switching and activation rates for a 593 nm emitter.* ***a.*** *PL spectra for an emitter that is activated under positive and negative bias.* ***b, c.*** *PL versus time traces where the emitter is activated repeatedly with +10 V (b) or -10 V (c) bias square pulse (0.5 s on then off).* ***d, e.*** *Emitter activation time under +10 V (d) and -10 V (e) bias, with τ_on_​ = 43 ± 0.8 ms and 8.3 ± 0.3 μs, respectively.*

**References**

1. Beveratos, A.; Kühn, S.; Brouri, R.; Gacoin, T.; Poizat, J,-P.; Grangier, P, Room temperature stable single-photon source. The European Physical Journal D-Atomic, Molecular, Optical and Plasma Physics. 2002, 18 (2) 191-6.

2. Kurtsiefer, C.; Mayer, S.; Zarda, P.; Weinfurter, H., Stable solid-state source of single photons. Physical review letters. 2000, 85 (2) 290.

3. Neu, E.; Steinmetz, D.; Riedrich-Möller, J.; Gsell, S.; Fischer, M.; Schreck, M.; Becher, C., Single photon emission from silicon-vacancy colour centres in chemical vapour deposition nano-diamonds on iridium. New Journal of Physics. 2011, 13 (2) 025012.

4. Babinec, T,-M.; Hausmann, B,-J.; Khan, M.; Zhang, Y.; Maze, J,-R.; Hemmer, P,-R.; Lončar, M.; A diamond nanowire single-photon source. Nature nanotechnology. 2010 5 (3) 195-9.
